# Characteristics of Two Crustins from *Alvinocaris longirostris* in Hydrothermal Vents

**DOI:** 10.3390/md19110600

**Published:** 2021-10-22

**Authors:** Lu-Lu Guo, Shao-Lu Wang, Fang-Chao Zhu, Feng Xue, Li-Sheng He

**Affiliations:** 1 Institute of Deep-Sea Science and Engineering, Chinese Academy of Sciences, Sanya 572000, China; guolulu112@idsse.ac.cn (L.-L.G.); wangshaolu2021@163.com (S.-L.W.); zhufc89@163.com (F.-C.Z.); 2 Institute of Clinical Pharmacology, Peking University, Beijing 100000, China; xuefeng2378@126.com

**Keywords:** crustins, antibacterial peptides, hydrothermal vent, anti-Gram-negative bacteria, Al-crus 3 and Al-crus 7

## Abstract

Crustins are widely distributed among different crustacean groups. They are characterized by a whey acidic protein (WAP) domain, and most examined Crustins show activity against Gram-positive bacteria. This study reports two Crustins, Al-crus 3 and Al-crus 7, from hydrothermal vent shrimp, *Alvinocaris longirostris*. Al-crus 3 and Al-crus 7 belong to Crustin Type IIa, with a similarity of about 51% at amino acid level. Antibacterial assays showed that Al-crus 3 mainly displayed activity against Gram-positive bacteria with MIC_50_ values of 10–25 μM. However, Al-crus 7 not only displayed activity against Gram-positive bacteria but also against Gram-negative bacteria Imipenem-resistant *Acinetobacter baumannii*, in a sensitive manner. Notably, in the effective antibacterial spectrum, Methicillin-sensitive *Staphylococcus aureus*, *Escherichia coli* (ESBLs) and Imipenem-resistant *A. baumannii* were drug-resistant pathogens. Narrowing down the sequence to the WAP domain, Al-crusWAP 3 and Al-crusWAP 7 demonstrated antibacterial activities but were weak. Additionally, the effects on bacteria did not significantly change after they were maintained at room temperature for 48 h. This indicated that Al-crus 3 and Al-crus 7 were relatively stable and convenient for transportation. Altogether, this study reported two new Crustins with specific characteristics. In particular, Al-crus 7 inhibited Gram-negative imipenem-resistant *A. baumannii*.

## 1. Introduction

Antimicrobial peptides (AMPs) are small molecular polypeptides with antibacterial activity, which is an important part of the innate immune system, especially for invertebrates, due to the lack of a specific immune system mediated by antibodies. Schnapp et al., first isolated an antimicrobial peptide from the blood cells of crab (*Carcinus maenas*) in 1996. This antimicrobial peptide is enriched with proline, with a molecular weight of about 6.5 kDa, and has antibacterial effects on Gram-positive and -negative bacteria [1]. Since then, research on antimicrobial peptides of crustacean has begun. The Crustin family is one of the most studied antimicrobial peptides from crustaceans [2,3,4].

The Crustin family is largely from crustaceans with a molecular weight of 7–14 kDa and is characterized by a four-disulfide core containing a whey acidic protein (WAP) domain located at the C-terminus, which is associated with multiple potential functions [5,6,7]. Over 50 Crustin sequences have been reported from various decapods, including crabs, lobsters, shrimp, and crayfish [5]. According to the characteristics of the sequences between the signal peptides and WAP domains, Crustins can be divided into four subtypes as follows. (1) type I: presence of a Cys-rich domain between the signal peptide and WAP domain; this type of antimicrobial peptide is mostly found in crabs, lobsters, and crayfish [8,9]. (2) type II Crustins, mainly found in shrimp, which have a Cys-rich domain and a Gly-rich region of about 40–80 amino acids (aa) adjacent to the signal peptide region [10,11]. They are usually active against Gram-positive bacteria and play a vital role in immune defense for crustaceans [12,13,14]. There are two sub-groups of type II Crustin (types IIa and IIb), initially classified by differences in the amino acid length of the Gly-rich region and the distance between the Cys-rich region and the WAP domain [15]. (3) Type III Crustins have neither a Gly-rich nor a Cys-rich region.Up to now, they are found only in *Penaeus monodon*, *Fenneropenaeus chinensis*, and *Eriocheir sinensis* [11,16,17]. Many researches do not classify type III Crustin and assign it to the general antimicrobial peptide, similar to Crustins. (4) Type IV Crustins possess two WAP domains and lack a Cys-rich domain [5]. The extra WAP domain at the C-terminal has other potential functions [18,19,20].

Although the antibacterial activities of Crustins have been widely reported, most are only against Gram-positive bacteria [21]. Only a few Crustins from *F. chinensiss*, spider crab, and *P. monodon* were reported as acting against Gram-negative bacteria with different activities. For example, CruHa1 from spider crab was found to act against Gram-negative bacteria, *Listonella anguillarum*, with a minimum inhibitory concentration (MIC) of 12.5 µM [22]. SpCrus6 from *Scylla Paramamosain* demonstrated weak activity against Gram-negative bacteria, including *Vibrio parahemolyticus*, *Vibrio alginolyticus*, *Vibrio harveyi*, and *Escherichia coli* with MIC >25 µM [2]. Additionally, CruHa1 and SpCrus6 belong to Crustin type I; a type II Crustin, Crus-like Pm, from *Penaeus monodon*, exhibited activity against Gram-negative bacteria with a MIC of 2.5–20 µM [11].

Deep-sea hydrothermal vents are chemosynthetic ecosystems and are extremely hot (200–400 °C), with high pH values and concentrations of heavy metal ions [23]. Due to their unusual chemical and physical features, hydrothermal vents are thought to house unique fauna; more than 600 animal species have been discovered in this extreme environment [24]. Furthermore, organisms living in this extreme environment have unique physiological and metabolic mechanisms to adapt to the extreme environment [25]. Recently, two Crustins, Re-Crustin (type II Crustin) and Crus1 (type I Crustin), were identified from the hydrothermal vent shrimp *Rimicaris exoculata* and *Rimicaris* sp. (Alvinocarididae family), respectively. Although Crus 1 and Re-Crustin shared a low sequence identity (24%) at the amino acid level, they both showed effective activity against Gram-positive bacteria with a MIC of 2.5–40 µM, but no activity against gram-negative bacteria [26,27].

This study characterized two Crustins (Al-crus 3 and Al-crus 7) from another vent shrimp of *Alvinocaris longirostris*. Furthermore, Al-crus 3 and Al-crus 7 were shown to have antibacterial activities on some pathogenic bacteria. Al-crus 7 demonstrated strong activity against imipenem-resistant *Acinetobacter baumannii*, gram-negative drug-resistant bacteria, with MIC_50_ at 12 µM. Thus, this study added new members to the Crustin family and showed that organisms living in extreme environments might contain unique antibacterial resources. 

## 2. Results

### 2.1. Characteristics of Al-crus 3 and Al-crus 7 Sequences

**Figure 1:**
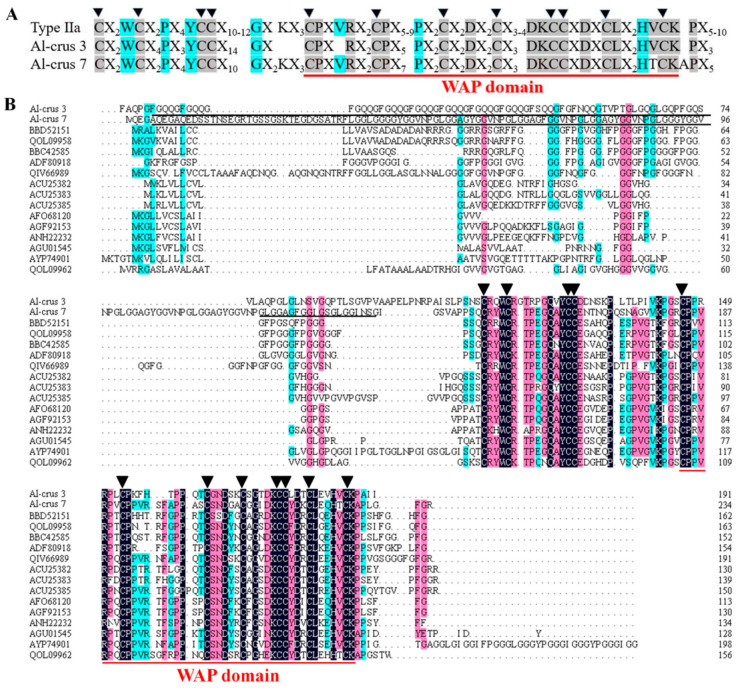
Comparison of amino acid sequences between Al-crus 3, Al-crus 7, and other Crustins. (**A**) Consensus amino acid sequence of Type IIa Crustins. X indicates any amino acid. Identical residues are highlighted. Triangles (▼) indicate the 12 conserved cysteine residues found in the Crustins. (**B**) Amino acid sequence alignments. Besides Al-crus 3 and Al-crus 7, the sequences used in this alignment were from *Penaeus vannamei* (QOL09958, QOL09962), *Panulirus japonicas* (ACU25382, ACU25383, BBC42585, BBD52151, AGU01545), *Macrobrachium rosenbergii* (ACU25385, AFO68120, AGF92153, ANH22232), *Penaeus paulensis* (ADF80918), *Macrobrachium nipponense* (QIV66989), and *Neocaridina heteropoda* (AYP74901). The Gly-rich domain is underlined by a solid black line, and the WAP domain is underlined by a solid red line. Triangles (▼) indicate the 12 conserved cysteine residues found in the Crustins, including the WAP domain.

Two Crustins, named Al-crus 3 and Al-crus 7, were cloned from the cDNA library of *Alvinocaris longirostris* with specific primers designed according to the annotations. The length of Al-crus 3 was 573 bp, with an ORF of 191 amino acids. Al-crus 3 contained a Gly-rich and WAP domain, which are shown by black and red lines, respectively, in Figure 1. The molecular weight (MW) of Al-crus 3 was 20 kDa with a theoretical *p*I of 7.98, which was calculated using ExPasy (https://web.expasy.org/, accessed on 21 September 2021). The length of Al-crus 7 was 702 bp, with an ORF of 234 amino acids. Al-crus 7 also contained Gly-rich and WAP domains (Figure 1). The theoretical *p*I and MW of mature Al-crus 7 were 6.44 and 22 kDa, respectively.

The deduced amino acid sequences of Al-crus 3 and Al-crus 7 were compared with those of other close Crustins (Figure 1). For Al-crus 3, the closest sequence was Crustin from *Macrobrachium nipponense* (NCBI GenBank accession no. QIV66989), with a similarity of 63% at the amino acid level. By contrast, for Al-crus 7, the closest sequence was a Crustin-like peptide from *Homarus americanus* (NCBI GenBank accession no. KAG7170693) with a similarity of 82% (Table S2). Based on the characteristics of the different Crustin types, Al-crus 3 and Al-crus 7 belonged to type IIa (Figure 1). There were eight conserved cysteine residues in the WAP domain and 12 cysteine residues in the C-terminal region. Among the 12 conserved cysteine residues, there were three amino acids between the first two cysteine residues (Cys_1_–Cys_2_), a sequence of 16 or 17 amino acids between Cys_4_–Cys_5_, and a sequence of 8–12 residues between Cys_6_–Cys_7_ (Figure 1). Thus, Al-crus 3 and Al-crus 7 shared around 51% amino acid sequences. Compared with the other two Crustins of Re-Crustin and Crus1 from other hydrothermal vent shrimps, the identities were 53% and 41% at the amino acid level for Al-crus 3, respectively. For Al-crus 7, the identities were 58% and 47%, respectively.

### 2.2. Phylogenetic Analysis of Al-crus 3 and Al-crus 7

**Figure 2:**
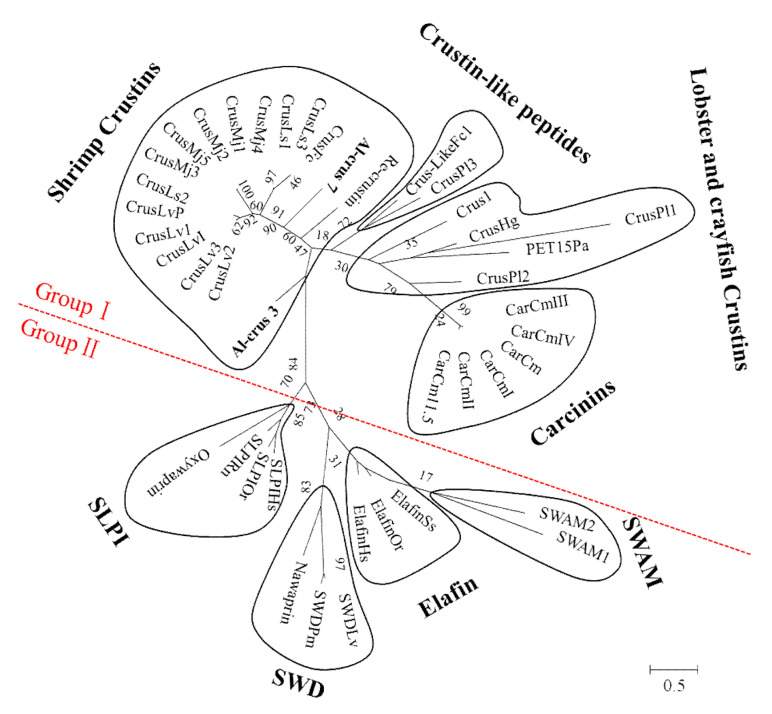
Unrooted phylogenetic tree constructed with Crustins from diverse sources. Crustins used in this analysis from diverse species included *Marsupenaeus japonicus* (Crus*Mj*1:AB121740; Crus*Mj*2: AB121741; Crus*Mj*3: AB121742; Crus*Mj*4: AB121743; Crus*Mj*5: AB121744), *Litopenaeus vannamei* (Crus*L*v1: AF430071; Crus*Lv*2: AF430072; Crus*Lv*3: AF430073; Crus*Lv*I: AY488492; Crus*Lv*P: AY488494), *L. setiferus* (Crus*Ls*1: AF430077; Crus*Ls*2: AF430078; Crus*Ls*3: AF430079), *Fenneropenaeus chinensi*s (CrusLike*Fc*1: DQ097703; Crus*Fc*: AY871268), *Carcinus maenas* (Car*Cm* 11.5: AJ237947; Car*Cm*: AJ427538; Car*Cm*-I: AJ821886; Car*Cm*-II: AJ821887; Car*Cm*-III: AJ821888; Car*Cm*-IV; AJ821889), *Homarus gammarus* (Crus*Hg*: CAH10349), *Pacifastacus leniusculus* (Crus*Pl*1: EF523612; Crus*Pl*2: EF523613; Crus*Pl*3: EF523614), *Panulirus argus* (PET15*Pa*: AAQ15293), *L. vannamei* (SWD*Lv*: AY465833), *P. monodon* (SWD*Pm*: AY464465), *Sus scrofa* (Elafin*Ss*: BAA08854), *Homo sapiens* (Elafin*Hs*: NP 002629), *Ovis aries* (Elafin*Or*: AAQ92320), *H. sapiens* (SLPI*Hs*: EAW75869), *O. aries* (SLPI*Or*: NP 001030302), *Rattus norvegicus* (SLPI*Rn*: AAN32722), *Naja nigricollis* (Nawaprin: P60589), *Oxyuranus microlepidotus* (Omwaprin: P83952), *Mus musculus* (SWAM1: AF276974 and SWAM2: AF276975), *Rimicaris* sp. (Crus 1: MW448473), and *Rimicaris exoculata* (*Re-Crustin:* MT102281). Values at the nodes indicate the percentage of times occurring in 1000 replications generated by bootstrapping the original deduced protein sequences. Al-crus 3 and Al-crus 7 are in bold.

WAP domain-containing proteins from diverse species were selected from NCBI for phylogenetic tree construction with Al-crus 3 and Al-crus 7. The results showed that these Crustins were mainly divided into two distinct groups: Group I and Group II. Furthermore, there were four clusters for each group (Figure 2); for Group I, the first cluster was shrimp Crustins. The Al-crus 3 and Al-crus 7 examined in this study were also classified into this cluster. Based on the Crustins present here, all the Crustins in this cluster were from shrimp. Some Crustins from shrimp were also classified into other clusters, such as CrusLike*Fc*1 from *Fenneropenaeus chinensis*, classified into the second cluster, Crustin-like peptides. Crus1 from *Rimicaris* sp. was clustered into the cluster of lobster and crayfish Crustins. The fourth cluster in Group I was made up of Carcinins, as they were all from *Carcinus maenas* based on the present Crustins. For Group II, the four clusters were SLPI, SWD, Elafins, and SWAM. The SWAM cluster included mouse single WAP motif protein 1 (SWAM1) and SWAM2 antibacterial proteins. 

### 2.3. Antibacterial Activities of Al-crus 3 and Al-crus 7

The recombinant Al-crus 3 and Al-crus 7 were expressed in *E. coli* BL21 (DE3), and the deduced molecular masses of the two recombinant proteins were 46 and 48 kDa, respectively, including 26 kDa of GST-tag. Seven Gram-positive bacteria and six Gram-negative bacteria were examined in this assay. The results showed that GST-Al-crus 3 mainly acted against Gram-positive bacteria, including *Micrococcus luteus*, *Bacillus subtilis*, *Staphylococcus aureus*, methicillin-sensitive *Staphylococcus aureus*, and *Escherichia coli* (ESBLs) with MIC_50_ values of 10–25 μM; whereas GST-Al-crus 3 showed almost no inhibitory activity against *Klebsiella Pneumoniae*, MRSA, and Gram-negative bacteria, up to 50 μM. Compared with GST-Al-crus 3, the recombinant GST-Al-crus 7 demonstrated an antibacterial spectrum that acted against Gram-positive bacteria, *Micrococcus luteus*, *Bacillus subtilis*, and methicillin-sensitive *Staphylococcus aureus*, and Gram-negative bacteria, imipenem-resistant *Acinetobacter baumannii*. However, GST-Al-crus 7 could barely inhibit the growth of other Gram-negative bacteria. Although GST-Al-crus 3 displayed strong activity against *S. aureus* with MIC_50_ of 10 μM, GST-Al-crus 7 revealed slight inhibitory activity against the growth of *S. aureus* (Table 1). Notably, methicillin-sensitive *S. aureus*, *E. coli* (ESBLs), and imipenem-resistant *A. baumannii* were drug-resistant pathogens in the effective antibacterial spectrum.

**Figure 3:**
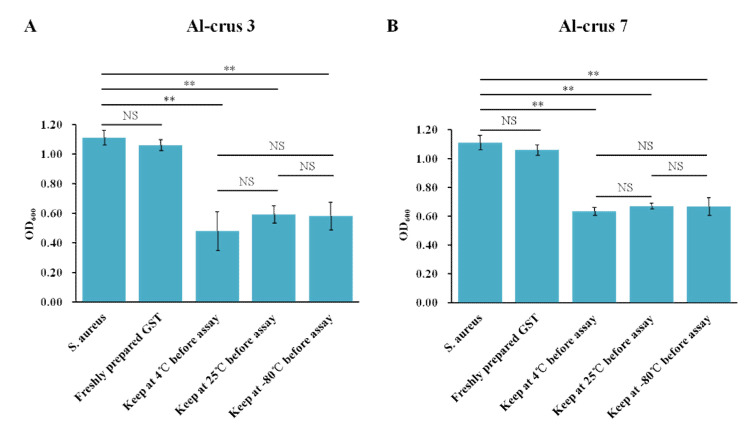
Thermal stabilities of GST-Al-crus 3 and GST-Al-crus 7. (**A**) *S. aureus* was treated with GST-Al-crus 3 for 12 h. Before the antibacterial assay, freshly purified GST-Al-crus 3 was kept at 4, 25, or −80 °C for 48 h, respectively. For control, GST was freshly purified. (**B**) *S.aureus* was incubated with GST-Al-crus 7 for 12 h. Before the antibacterial assay, freshly purified GST-Al-crus 7 was kept at 4, 25, or −80 °C for 48 h. For control, GST was freshly purified. Values are shown as means ± SD (standard deviation; N ≥ 3). Asterisks show significant differences between Crustin-treated samples and control. **: *p* < 0.01; NS, not significant (one-way ANOVA).

To evaluate the thermal stability of Al-crus 3 and Al-crus 7, the GST-Al-crus 3 and GST-Al-crus 7 were kept at different temperatures for 48 h, and then an antibacterial assay was performed on *S. aureus*. The results showed that there were no significant differences for GST-Al-crus 3 against *S. aureus* after kept at 4, 25, or −80 °C for 48 h, which was also true for GST-Al-crus 7 (Figure 3).

To further investigate whether the WAP domain is enough for Crustins to act against bacteria, two peptides containing the WAP domain from Al-crus 3 and Al-crus 7, designed as Al-crusWAP 3 and Al-crusWAP 7, were chemically synthesized, respectively. Al-crusWAP 3 displayed the same effect as Al-crus 3 on *Micrococcus luteus* and *Bacillus subtilis*. However, for *Staphylococcus aureus*, methicillin-sensitive *Staphylococcus aureus* and *Escherichia coli* (ESBLs), higher MIC_50_ values were needed compared with that of Al-crus 3. For Al-crusWAP 7, the effects on *Micrococcus luteus* and methicillin-sensitive *Staphylococcus aureus* were the same as Al-crus 7. However, the MIC_50_ of the antibacterial assays on *Bacillus subtilis* and imipenem-resistant *Acinetobacter baumannii* resulted in higher values. These results revealed that although Al-crusWAP 3 and Al-crusWAP 7 demonstrated antibacterial activity, the effect was weaker than that of the full-length of Al-crus 3 and Al-crus 7 (Table 1).

### 2.4. SEM Imaging

**Figure 4:**
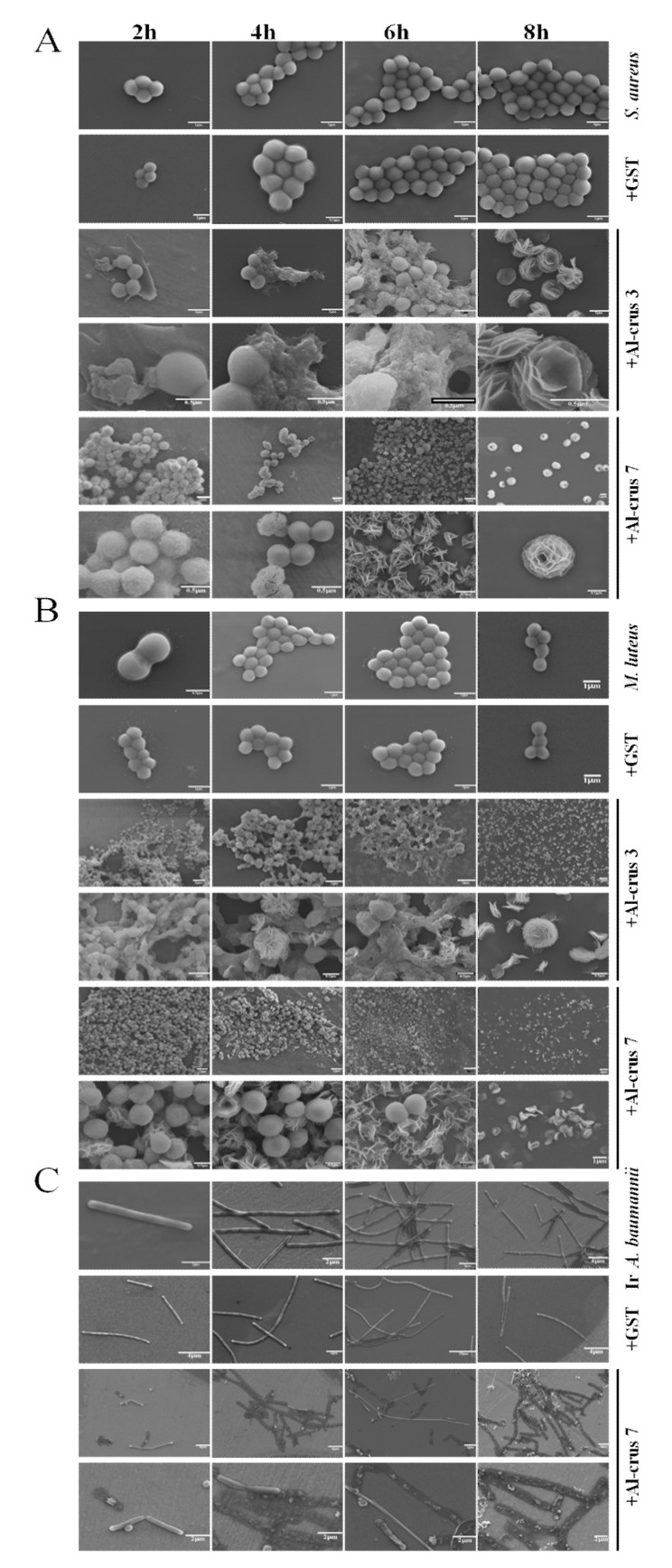
Images of the cells treated with GST-Al-crus 3 and GST-Al-crus 7 at different periods. (**A**) Images of *S. aureus* were observed at 2, 4, 6, and 8 h after treatment with GST-Al-crus 3 and GST-Al-crus 7. GST was used as a control. (**B**) Images of *M. luteus* were observed at 2, 4, 6, and 8 h after treatment with Al-crus 3 and Al-crus 7. GST was used as a control. (**C**) Images of imipenem-resistant *Acinetobacter baumannii* were observed at 2, 4, 6, and 8 h after treatment with Al-crus 7. GST was used as a control. IR: imipenem-resistant.

The images of the cells were observed using a SEM apparatus after treatment with GST-Al-crus 3 and GST-Al-crus 7. *S. aureus, M. luteus*, and imipenem-resistant *A. baumannii* were used as examples. The results showed that after treatment for 2 h, the cells underwent morphological changes. Specifically, during the treatment of GST-Al-crus 3, the cell membranes of *S. aureus* and *M. luteus* were ruptured and the cell contents leaked; during the treatment of GST-Al-crus 7, the membranes of *S. aureus* became more permeable and the membranes of *M. luteus* became wrinkled. After treatment for 4 h, the number of damaged cells increased. Almost all the examined cells showed morphological changes or were broken after a 6 h treatment (Figure 4). Notably, for *S. aureus* and *M. luteus*, although the cell morphologies changed after treatment with GST-Al-crus 3 and GST-Al-crus 7, their changes were different (Figure 4). By comparison, the cells did not show any change after GST treatment (Figure 4). 

## 3. Discussion

Marine organisms are a promising reservoir of bioactive products for drug discovery. Additionally, market analysis forecasts that the global market for marine-derived drugs is expected to reach USD 2745.80 million by 2025 [28]. However, to date, few molecular compounds from marine organisms have been approved and applied in clinics. Furthermore, many marine ecosystems have not been explored, especially extreme environments. Extreme environments feature one or more parameters, such as temperature, salinity, osmolality, UV radiation, pressure, and pH, that show values close to the limit of life. Marine organisms living in extreme environments adopt unique survival strategies for survival and reproduction, biosynthesizing an array of biomolecules that are potentially valuable for many applications, such as biotechnology and pharmaceutics. Hydrothermal vents are extreme environments in the deep sea with high salinity, pressure, and temperature, usually on the ocean floor, such as mid-ocean ridges, where tectonic plates are pulled apart. Although an increasing body of research has been conducted on microbiota from hydrothermal vents, there are few studies on macroorganisms. There is even less research on active molecules derived from hydrothermal vent macroorganisms. For example, there are only two published papers related to Crustins from hydrothermal vent macroorganisms. One study reported that a type I Crustin, Crus1, was identified from a hydrothermal vent shrimp, *Rimicaris* sp. Crus1 shared the highest identity, around 51%, with a type I Crustin from *Penaeus vannamei*. Crus1 demonstrated effective activity against Gram-positive bacteria by binding to the peptidoglycan and lipoteichoic acid of the target cell membrane [26]. Another published study analyzed a type II Crustin (Re-Crustin) from hydrothermal vent *R. exoculata*, which displayed activity against Gram-positive bacteria. In this study, two type IIa Crustins, Al-crus 3 and Al-crus 7, from *Alvinocaris longirostris* were identified and characterized. Al-crus 7 demonstrated activity against some Gram-positive bacteria and one Gram-negative bacterium in this study. Furthermore, Al-crus 3 and Al-crus 7 affected some drug-resistant pathogens. These results reveal the potential of bioactive molecules from hydrothermal vent macroorganisms. The analysis of the phylogenetic tree indicated that the four vent Crustins were classified into different clusters. Crus 1 was classified into lobster and crayfish Crustins and the other three were in shrimp Crustins, although all of the four Crustins were from vent shrimp. Similar phenomena were observed in some other Crustins, such as CrusLike*Fc*1 and Crus*Fc*; although both from *Fenneropenaeus chinensi*s, they were assigned to different clusters. Crus*Pl*1, Crus*Pl*2 and Crus*Pl*3 are from *Pacifastacus leniusculus*, but unlike Crus*Pl*1, Crus*Pl*2, Crus*Pl*3 was assigned to the cluster of Crustin-like peptides. These results suggested that besides the phylogenetic relationships between these macroorganisms, environment microorganisms might be also involved in the evolution of these Crustins.

Antimicrobial peptides are small molecular polypeptides with antibacterial activities that widely exist in organisms, and represent an important part of the body’s innate immune system. When pathogenic microorganisms infect the body, they can be synthesized rapidly. When the body produces an inflammatory response, AMPs are generated and released. Furthermore, AMPs are an important molecular barrier for the host to defend against the invasion of pathogenic microorganisms [29]. Antimicrobial peptides have the advantages of low molecular weight, good water solubility, thermal stability, and nontoxicity to the normal cells of higher animals [30]. Moreover, they are easily degraded and cannot easily produce residues. They exhibit different antibacterial mechanisms from antibiotics and can be considered as new anti-bacterial reagents replacing antibiotics. Until now, more than ten antimicrobial peptide families have been found. Furthermore, there are three main AMPs in crustaceans: Penaeidins, Crustins, and anti-lipopolysaccharide factor [2,3,4]. Antibacterial peptides are highly diverse, except for those derived from highly conserved protein cleavage; different species have specific antimicrobial peptide sequences; even species that are closely related are not exempt. There are seven to dozens of antibacterial peptides in each organism [3,31]. Antibacterial peptides exhibit a broad spectrum of antibacterial activity against Gram-positive and -negative bacteria, fungi, and viruses. However, the antibacterial spectrum of each antibacterial peptide is different [32]. In this study, two Crustins were characterized. Although Al-crus 3 and Al-crus 7 were from the same species and belonged to type IIa Crustins, they shared a similar sequence of only about 51% at the amino acid level and displayed different antibacterial activities. Al-crus 3 only displayed inhibitory activity against Gram-positive bacteria, but Al-crus 7 displayed it against some Gram-positive bacteria and one Gram-negative bacterium in this study. Even in the activity against -Gram-positive bacteria, their antibacterial spectrum was different. For Al-crus 3, the Gram-positive bacteria against which they acted encompassed *Micrococcus luteus*, *Bacillus subtilis*, *Staphylococcus aureus*, methicillin-sensitive *Staphylococcus aureus*, and *Escherichia coli* (ESBLs). However, Al-crus 7 only inhibited *Micrococcus luteus*, *Bacillus subtilis*, methicillin-sensitive *Staphylococcus aureus*, and *Escherichia coli* (ESBLs). By contrast, Al-crus 7 inhibited imipenem-resistant *Acinetobacter baumannii* with MIC_50_ of 12 µM. The diversity of antimicrobial peptides and their functions are related to the host’s response to various pathogenic bacteria and the adjustment of symbiotic flora. 

For Crustins, the sequence feature contained at least one WAP domain at their C-terminus. This domain has eight cysteine residues in a conserved arrangement that forms a tightly packed structure, described on PROSITE as a four-disulfide core (4DSC). Previous studies suggest that the antibacterial activity of Crustins is related to the WAP domain. Comparing CruFc with the WAP domain from *Fenneropenaeus chinensis*, which produces strong antibacterial activity against Gram-positive bacteria, CshFc without the WAP domain has almost no antibacterial activity [26]. After mutating the eight Cys residues in the WAP domain of rCrus1 from the deep-sea hydrothermal vent, none of the mutants exhibited bactericidal activity at the minimum bactericidal concentration of rCrus2 [26]. These results supported the viewpoint that the WAP domain is important for the antibacterial activities of Crustins. Nevertheless, no published report has shown whether the WAP domain is enough for Crustins to perform their activities. This study synthesized two peptides, Al-crusWAP 3 and Al-crusWAP 7, derived from Al-crus 3 and Al-crus 7, with only the WAP domain. Apart from *Micrococcus luteus* and *Bacillus subtilis*, Al-crusWAP 3 displayed effects against *Staphylococcus aureus*, methicillin-sensitive *Staphylococcus aureus*, and *Escherichia coli* (ESBLs) with higher MIC_50_ values compared with that of Al-crus 3. Additionally, Al-crusWAP 7 demonstrated the same effects on *Micrococcus luteus* and methicillin-sensitive *Staphylococcus aureus*, compared with Al-crus 7. However, for *Bacillus subtilis* and imipenem-resistant *Acinetobacter baumannii*, Al-crusWAP 7 displayed a higher MIC_50_ value. These results showed that the two peptides exhibited lower antibacterial activities than Al-crus 3 and Al-crus 7, respectively, thus suggesting that other amino acid sequences can contribute together with the WAP domain to the observed antibacterial activity. 

## 4. Materials and Methods

### 4.1. Strains, Vectors, Reagents, and Enzymes

The bacteria tested in this study, including *Micrococcus luteus* (NRR00100), *Bacillus subtilis* (NRR00591), *Staphylococcus aureus* (NRR01280), and *Salmonella* sp. (NRR00490), were obtained from Huayueyang Biotech Co., Ltd., Beijing, China. The drug-resistant bacteria included the Gram-positive bacteria, *Klebsiella Pneumoniae* (ESBLs, extended spectrum beta-lactamases; Store No. 0244), methicillin-resistant *Staphylococcus aureus* (MRSA; Store No. H57), methicillin-sensitive *Staphylococcus aureus* (Store No. G280), *Escherichia coli* (ESBLs, Store No. G160); and the Gram-negative bacteria, imipenem-sensitive *Pseudomonas aeruginosa* (Store No. E248), imipenem-resistant *Acinetobacter baumannii* (Store No. E292), imipenem-sensitive *Acinetobacter baumannii* (Store No. H422), *Klebsiella Pneumoniae* (ESBLs, Store No. F161), and *Escherichia coli* (ESBLs, Store No. K8). All were obtained from the Institute of Clinical Pharmacology, Peking University, Beijing, China. The aforementioned bacteria were kept at −80 °C with 20% glycerinum until use. The *E. coli* host strain BL21 (DE3) chemically competent cell was obtained from TransGen Biotech (Beijing, China). Additionally, the vector pGEX-4T-1 was obtained from Qiagen (Hilden, German) and the vector pMD 18-T and Taq DNA polymerase were obtained from Takara (Dalian, China). The GST-sefinose (TM) resin was obtained from Sangon (Shanghai, China). Finally, the ampicillin, chloramphenicol, and IPTG were purchased from Sigma (Guangzhou, China), the bacterial culture components were obtained from Sigma (Guangzhou, China), and the restriction enzymes were obtained from Takara (Dalian, China).

### 4.2. Gene Cloning of Al-crus 3 and Al-crus 7

The RNA extraction, sequencing, assembly, and annotation were performed according to our laboratory’s published paper [33]. Based on the sequences of the annotated Crustins, two paired primers of Crustins, Al-crus 3 and Al-crus 7, were designed (Table S1). The cDNA library for cloning was synthesized using PrimeScript II 1st Strand cDNA Synthesis kit (Dalian, China). Briefly, a 10 µL reaction containing 1 µL Oligo dT Primer (50 µM), 1 µL dNTP mixture (10 mM), and 5 µg total RNA and RNase-Free dH_2_O were kept at 65 °C for 5 min, and then immediately cooled on ice. Next, a 20 µL reaction mixture was prepared by combining the following reagents: 10 µL template RNA and primer mixture (from above), 4 µL 5 × PrimeScript Buffer, 20 units RNase inhibitor, 200 units PrimeScript II RTase, and 4.5 µL RNase-free dH_2_O. After being gently mixed, the reaction mixture was incubated immediately at 42 °C for 45 min and then incubated at 95 °C for 5 min to inactivate the enzymes; this was followed by cooling down on ice. For the targeted Crustin amplification, a 50 µL reaction containing 1 µL of the previously prepared cDNA, 10 µL 5 × PCR buffer, 4 µL of 10 mM dNTPs, 0.5 µL Primer STAR HS DNA Polymerase (Takara, Japan), 32.5 µL ddH_2_O, and 2 µL of 10 uM for each primer was prepared. The PCR program consisted of an initial step of denaturation at 98 °C for 10 s, followed by 30 cycles of 98 °C for 10 s, 50 °C for 30 s, and 72 °C for 1 min, with a final extension of 10 min at 72 °C. The PCR products were purified and linked into the pMD 18-T vector and transferred into the DH5α competent cells. After being cultured at 37 °C overnight with ampicillin, positive colonies were obtained and identified by sequencing (BGI, Shenzhen, China). 

### 4.3. Sequence Alignment

A Basic Local Alignment Search Tool (BLAST) in NCBI server was used to perform the sequence comparison with the GenBank protein database. The sequences of different WAP domain-containing proteins with high similarity were selected from NCBI and are listed in Supplementary Table S2. The sequence alignment was constructed using ClustalW (v.2.0), and a phylogenetic tree was created using the maximum likelihood model of MEGA (v.6.0) with 1000 replications. 

### 4.4. Plasmids, Expression, and Purification of Al-crus 3 and Al-crus 7

Al-crus 3 and Al-crus 7 were cloned into a pGEX4T-1 vector with the restriction enzymes *Kpn* and *EcorR*I. The procedures of ligation, colony selection, and sequencing were similar to the above mentioned. After the sequence identification, GST-Al-crus 3 and GST-Al-crus 7 were expressed by transferring them into *Escherichia coli* BL21(DE3) cells and then purified by affinity chromatography using GenScript High-Affinity GST Resin, following the manufacturer’s protocol (Sangon, Shanghai, China). Briefly, the *E. coli* BL21(DE3) with recombinant plasmid was cultured at 37 °C in lysogeny broth (LB) containing 100 µg/ml ampicillin and 50 µg/ml chloromycetin for 12 h. The cultures were diluted (1:1000) with LB broth and subjected to further incubation until the OD_600_ reached about 0.8, and then induced by isopropyl β-D-thiogalactoside (IPTG) at a final concentration of 0.5 mM. After induction for 12 h at 28 °C, the cells were collected and broken by an ultrasonic binding/wash buffer (1 × PBS with 1% Triton X-100) at 4 °C. After ultrasonication, the cell debris was removed by centrifugation at 8000× *g* for 30 min, and the supernatant was retained. The recombinant proteins were purified directly from the lysate using GST-sefinose (TM) resin. The supernatant was applied to a Poly-Prep Chromatography Column (BIO-RAD, USA) with 1 ml GST-sefinose (TM) resin, which was pre-washed with a binding/washing buffer. The purified proteins were dialyzed in 1 × PBS at 4 °C for 24 h, with the 1 × PBS replaced every 12 h. The protein concentration was determined using the Bradford method, using BSA (bovine serum albumin) as the standard. The purified proteins were mixed with a 6 × SDS gel-loading buffer, boiled at 100 °C for 10 min, and resolved with 12% sodium dodecyl sulfate-polyacrylamide gel electrophoresis (SDS-PAGE). The gels were stained with Coomassie brilliant blue R250. Finally, the purified proteins were stored at −80 °C in aliquots, unless otherwise specified.

### 4.5. Peptide Synthesis

The peptides from Al-crus 3 and Al-crus 7 containing the WAP domain were designed and synthesized by GenScript Biological Technology Co., LTD. Al-crusWAP-3 from Al-crus 3: SCPPRRPLCPKFHTPPQTCGNDSKCSGTDKCCLDTCLEVCK, and Al-crusWAP 7 from Al-crus 7: RCPPVRPVCPPVRSFAPPASCSNDGACGGIDKCCYDKCLEQHTCK. The purity of these peptides was more than 98%.

### 4.6. Antibacterial Activity Assays

The examined bacteria from the −80 °C stock were first inoculated on plates, and then a single colony for culture was picked up in LB broth. To avoid contamination, the tested bacteria were further sequenced and identified. Antimicrobial activities were examined against seven Gram-positive and six Gram-negative bacteria. The MIC was determined by a liquid growth inhibition assay [34]. The purified proteins were consecutively diluted with sterile water in five multiples; next, 0.2% BSA was used as the negative control. Aliquots (10 μL) from each dilution were transferred to a 96-well polypropylene microtiter plate (Corning, Wujiang, China), and each well was inoculated with 100 μL of mid-log bacterial suspension (10^5^ CFU/ml) in poor broth (1% tryptone, 0.5% NaCl (*w*/*v*), pH 7.5). The experimental assays were grown for 12 h with shaking at 120 rpm/hr and 37 °C. The OD_600_ values were measured every 4 h using a microplate reader (Multiscan FC, Thermo Fisher, American). All the experiments were performed at least three times for the replications. For the thermal stability analysis, the freshly purified proteins were kept at different temperatures for 48 h and then processed to perform antibacterial assays, as mentioned above. 

For the peptide antimicrobial activity experiment, the bacteria were the same as those mentioned above. The peptides were centrifuged before dissolution with ddH_2_O to 550 µM and kept at −80 °C in aliquots. Finally, the MIC_50_ was determined.

### 4.7. SEM Imaging

The *M. luteus*, *S. aureus*, and imipenem-resistant *Acinetobacter baumannii* were treated with Al-crus 3 and Al-crus 7 with a MIC_50_ concentration, respectively. The treated and controlled samples were collected at 2, 4, 6, and 8 h, respectively. After being washed with PBS, the cells were resuspended in a PBS buffer to about 1 × 10^6^ CFU/ml. Next, the cells were fixed with 4% PFA, 5 μL of which were added to the copper films for incubation overnight. After drying, the cooper film with the cells was examined with SEM (JSM-7100F, JEOL, Beijing, China). The normal and abnormal cells were photographed.

## Figures and Tables

**Table 1:** Antibacterial activities of Al-crus 3, Al-crus 7, and their deduced WAP domains.

Microorganism	Store No.	MIC_50_(μM)			
		Al-crus 3	Al-crusWAP 3	Al-crus 7	Al-crusWAP 7
**Gram-positive bacteria**					
*Micrococcus luteus*	NRR00100	25	25	10	10
*Klebsiella Pneumoniae* (ESBLs) *	0244	>50	>50	>50	>50
*Bacillus subtilis*	NRR00591	25	25	8	25
*Staphylococcus aureus*	NRR01280	10	25	50	>50
Methicillin-resistant *Staphylococcus aureus* *	H57	>50	>50	>50	>50
Methicillin-sensitive *Staphylococcus aureus* *	G280	10	25	25	25
*Escherichia coli* (ESBLs) *	G106	25	>50	>50	>50
**Gram-negative bacteria**					
*Escherichia coli* (ESBLs) *	K8	>50	>50	>50	>50
Imipenem-sensitive *Pseudomonas aeruginosa* *	E248	>50	>50	>50	>50
Imipenem-resistant *Acinetobacter baumannii* *	E292	>50	>50	12	>50
Imipenem-sensitive *Acinetobacter baumannii* *	H422	>50	>50	>50	>50
*Klebsiella Pneumoniae* (ESBLs) *	F161	>50	>50	>50	>50
*Salmonella* sp.	NRR00490	>50	>50	>50	>50

* Means drug-resistant pathogenic bacteria.

## Data Availability

The original contribution presented in this study are included in the article/Supplementary Material, further inquiries can be directed to the corresponding author.

## Supplementary Materials

The following are available online at https://www.mdpi.com/article/10.3390/md19110600/s1, Table S1: Primers with restriction enzymes used for cloning, Table S2: The similarities between Al-crus 3, Al-crus 7 and WAP domain-containing protein/peptides in crustaceans.

## References

[1] Schnapp D., Kemp G.D., Smith V.J. (1996). Purification and characterization of a proline-rich antibacterial peptide, with sequence similarity to bactenecin-7, from the haemocytes of the shore crab, Carcinus maenas. Eur. J. Biochem..

[2] Du Z.Q., Wang Y., Ma H.Y., Shen X.L., Wang K., Du J., Yu X.D., Fang W.H., Li X.C. (2019). A new crustin homologue (SpCrus6) involved in the antimicrobial and antiviral innate immunity in mud crab, Scylla paramamosain. Fish Shellfish Immun..

[3] Jiang H.S., Jia W.M., Zhao X.F., Wang J.X. (2015). Four crustins involved in antibacterial responses in Marsupenaeus japonicus. Fish Shellfish Immun..

[4] Hoffmann J., Schneider C., Heinbockel L., Brandenburg K., Reimer R., Gabriel G. (2014). A new class of synthetic anti-lipopolysaccharide peptides inhibits influenza A virus replication by blocking cellular attachment. Antivir. Res..

[5] Smith V.J., Fernandes J.M.O., Kemp G.D., Hauton C. (2008). Crustins: Enigmatic WAP domain-containing antibacterial proteins from crustaceans. Dev. Comp. Immunol..

[6] Hauton C., Brockton V., Smith V.J. (2006). Cloning of a crustin-like, single whey-acidic-domain, antibacterial peptide from the haemocytes of the European lobster, Homarus gammarus, and its response to infection with bacteria. Mol. Immunol..

[7] Sallenave J.-M. (2000). The role of secretory leukocyte proteinase inhibitor and elafin (elastase-specific inhibitor/skin-derived antileukoprotease) as alarm antiproteinases in inflammatory lung disease. Respir. Res..

[8] Brockton V., Hammond J.A., Smith V.J. (2007). Gene characterisation, isoforms and recombinant expression of carcinin, an antibacterial protein from the shore crab, Carcinus maenas. Mol. Immunol..

[9] Jiravanichpaisal P., Lee S.Y., Kim Y.-A., Andrén T., Söderhäll I. (2007). Antibacterial peptides in hemocytes and hematopoietic tissue from freshwater crayfish Pacifastacus leniusculus: Characterization and expression pattern. Dev. Comp. Immunol..

[10] Rosa R.D., Bandeira P.T., Barracco M.A. (2007). Molecular cloning of crustins from the hemocytes of Brazilian penaeid shrimps. Fems. Microbiol. Lett..

[11] Amparyup P., Kondo H., Hirono I., Aoki T., Tassanakajon A. (2008). Molecular cloning, genomic organization and recombinant expression of a crustin-like antimicrobial peptide from black tiger shrimp Penaeus monodon. Mol. Immunol..

[12] Shockey J., O’leary N.A., De La Vega E., Browdy C.L., Baatz J.E., Gross P.S. (2009). The role of crustins in Litopenaeus vannamei in response to infection with shrimp pathogens: An in vivo approach. Dev. Comp. Immunol..

[13] Hipolito S.G., Shitara A., Kondo H., Hirono I. (2014). Role of Marsupenaeus japonicus crustin-like peptide against Vibrio penaeicida and white spot syndrome virus infection. Dev. Comp. Immunol..

[14] Sun B., Wang Z., Zhu F. (2017). The crustin-like peptide plays opposite role in shrimp immune response to Vibrio alginolyticus and white spot syndrome virus (WSSV) infection. Fish Shellfish Immun..

[15] Tassanakajon A., Somboonwiwat K., Amparyup P. (2015). Sequence diversity and evolution of antimicrobial peptides in invertebrates. Dev. Comp. Immunol..

[16] Zhang J., Li F., Wang Z., Xiang J. (2007). Cloning and recombinant expression of a crustin-like gene from Chinese shrimp, Fenneropenaeus chinensis. J. Biotechnol..

[17] Mu C.K., Zheng P.L., Zhao J.M., Wang L.L., Qiu L.M., Zhang H., Gai Y.C., Song L.S. (2011). A novel type III crustin (CrusEs2) identified from Chinese mitten crab Eriocheir sinensis. Fish Shellfish Immun..

[18] Jia Y.P., Sun Y.D., Wang Z.H., Wang Q., Wang X.W., Zhao X.F., Wang J.X. (2008). A single whey acidic protein domain (SWD)-containing peptide from fleshy prawn with antimicrobial and proteinase inhibitory activities. Aquaculture.

[19] Supungul P., Klinbunga S., Pichyangkura R., Hirono I., Aoki T., Tassanakajon A. (2004). Antimicrobial peptides discovered in the black tiger shrimp Penaeus monodon using the EST approach. Dis. Aquat. Organ..

[20] Rojtinnakorn J., Hirono I., Itami T., Takahashi Y., Aoki T. (2002). Gene expression in haemocytes of kuruma prawn, Penaeus japonicus, in response to infection with WSSV by EST approach. Fish Shellfish Immun..

[21] Imjongjirak C., Amparyup P., Tassanakajon A., Sittipraneed S. (2009). Molecular cloning and characterization of crustin from mud crab Scylla paramamosain. Mol. Biol. Rep..

[22] Sperstad S.V., Haug T., Paulsen V., Rode T.M., Strandskog G., Solem S.T., Styrvold O.B., Stensvag K. (2009). Characterization of crustins from the hemocytes of the spider crab, Hyas araneus, and the red king crab, Paralithodes camtschaticus. Dev. Comp. Immunol..

[23] Little C.T.S., Vrijenhoek R.C. (2003). Are hydrothermal vent animals living fossils?. Trends Ecol. Evol..

[24] Van Dover C.L., German C.R., Speer K.G., Parson L.M., Vrijenhoek R.C. (2002). Marine biology-Evolution and biogeography of deep-sea vent and seep invertebrates. Science.

[25] Hazel J.R., Williams E.E. (1990). The Role of Alterations in Membrane Lipid-Composition in Enabling Physiological Adaptation of Organisms to Their Physical-Environment. Prog. Lipid. Res..

[26] Wang Y., Zhang J., Sun Y., Sun L. (2021). A Crustin from Hydrothermal Vent Shrimp: Antimicrobial Activity and Mechanism. Mar. Drugs.

[27] Bloa S.L., Boidin-Wichlacz C., Cueff-Gauchard V., Rosa R.D., Tasiemski A. (2020). Antimicrobial Peptides and Ectosymbiotic Relationships: Involvement of a Novel Type IIa Crustin in the Life Cycle of a Deep-Sea Vent Shrimp. Front. Immunol..

[28] (2021). Marine Derived Drugs Market Size By Type (Phenol, Steroid, Ether, Peptide), By Source (Algae, Microorganisms, Invertebrates), By Application (Anti-microbial, Anti-viral, Anti-inflammatory, Anti-tumor, Anti-cardiovascular, Other), By Region (North America, Europe, Asia-Pacific, Rest of the World), Market Analysis Report, Forecast 2021–2026. Pharm. Am. Market Res. Engine.

[29] Wang G.S., Watson M.W., Buckheit R.W. (2008). Anti-human immunodeficiency virus type 1 activities of antimicrobial peptides derived from human and bovine cathelicidins. Antimicrob. Agents Chemother..

[30] Bulet P., Stocklin R., Menin L. (2004). Anti-microbial peptides: From invertebrates to vertebrates. Immunol. Rev..

[31] Donpudsa S., Visetnan V., Supungul P., Tang S., Tassanakajon A., Rimphanitchayakit V. (2014). Type I and type II crustins from Penaeus monodon, genetic variation and antimicrobial activity of the most abundant crustinPm4. Dev. Comp. Immunol..

[32] Krusong K., Poolpipat P., Supungul P., Tassanakajon A. (2012). A comparative study of antimicrobial properties of crustinPm1 and crustinPm7 from the black tiger shrimp Penaeus monodon. Dev. Comp. Immunol..

[33] Zhu F.C., Sun J., Yan G.Y., Huang J.M., Chen C., He L.S. (2020). Insights into the strategy of micro-environmental adaptation: Transcriptomic analysis of two alvinocaridid shrimps at a hydrothermal vent. PLoS ONE.

[34] Roncevic T., Cikes-Culic V., Maravic A., Capanni F., Gerdol M., Pacor S., Tossi A., Giulianini P.G., Pallavicini A., Manfrin C. (2020). Identification and functional characterization of the astacidin family of proline-rich host defence peptides (PcAst) from the red swamp crayfish (Procambarus clarkii, Girard 1852). Dev. Comp. Immunol..

