# Supplementary material for: Characteristics of Two Crustins from Alvinocaris longirostris in Hydrothermal Vents
Source: Mar Drugs. 2021 Oct 22;19(11):600. doi: 10.3390/md19110600 (PMC8626000; doi:10.3390/md19110600)
Supplement: Supplementary file 1 [file marinedrugs-19-00600-s001.zip › marinedrugs-1423485-supplementary.pdf]

Table S1. Primers with restriction enzymes used for cloning

Gene	Forward Primer (5′-3′)	Reverse Primer (5′-3′)
Al-crus 3	TTCGCCCAACCAGGATTCGG	TGTGCAAACCTGCCATCATATAA
Al-crus 7	ATGCAAGAAGGTGCTCAAG	ACTTGGCTTCGGGCGTTAA

Table S2. The similarities between Al-crus 3, Al-crus 7 and WAP domain-containing protein/peptides in crustaceans

Species		Accession no.	identity	
			Al-crus 3	Al-crus 7
Palaemonidae	*Macrobrachium nipponense*	QIV66989	63%	56%
		QEQ76263	52%	63%
	Macrobrachium rosenbergii	AFO68120	-	67%
		AGF92153	-	66%
		ANH22232	-	60%
		ACU25385	59%	64%
	Panulirus japonicus	ACU25382	59%	63%
		ACU25383	57%	-
		AGU01545	54%	66%
		BBD52151	-	63%
		BBC42585	-	63%
	Penaeus vannamei	QOL09968	59%	65%
		ROT79125	59%	-
		AAL36891	57%	-
		QOL09962	56%	68%
		AAL36892	55%	58%
		ROT79124	55%	56%
		AAS59735	55%	-
		AAL36894	55%	-
		AAL36893	54%	-
		AAL36895	54%	-
		AYM00403	52%	-
		QOL09958	-	64%
	Penaeus paulensis	ABM63361	57%	58%
		ADF80918	-	58%
	Penaeus subtilis	ABO93323	55%	58%
	Penaeus setiferus	AAL36897	55%	-
	Panulirus brasiliensis	ABQ96197	54%	59%
	Penaeus schmitti	ABM63362	54%	-
Nephropidae	*Neocaridina heteropoda*	AYP74901	56%	58%
	Homarus americanus	KAG7170693	-	82%
